# Soil Seed Banks and Their Relation to Soil Properties in Hilly Landscapes

**DOI:** 10.3390/plants13010104

**Published:** 2023-12-29

**Authors:** Regina Skuodienė, Vilija Matyžiūtė, Gintaras Šiaudinis

**Affiliations:** Lithuanian Research Centre for Agriculture and Forestry Vezaiciai Branch, Gargzdu Str. 29, Klaipeda District, LT-96216 Vezaiciai, Lithuania; vilija.matyziute@lammc.lt (V.M.); gintaras.siaudinis@lammc.lt (G.Š.)

**Keywords:** hill, seed bank, soil runoff sediments, agrophytocenoses, edaphic factors

## Abstract

For the prevention of hilly soils from erosion, a smart selection of crop rotations is very important. The aim of this study was to investigate the influence of different agrophytocenoses on seed numbers in the soil runoff sediments and soil seed banks’ relations to soil properties in hilly landscapes. This study analyzes long-term monitoring data from three different agrophytocenoses (permanent grassland, cereal–grass crop rotation and crop rotation with a row crop) set up on slopes of 9–11° steepness with collectors for soil and water installed. The soil of the southern exposition slope was a slightly eroded Eutric Retisol. In the soil of permanent grassland, the number of seeds was 4036 seeds m^−2^, 6.0 and 3.2 times smaller compared to cereal–grass crop rotation and crop rotation with a row crop. The seeds found in the soil runoff sediments composed, on average, 0.9% of the soil seed bank, and the number of seeds depended on the number of days with heavy precipitation during the plant vegetation period, as well as on the plant communities grown in a particular rotation. Correlation analysis showed the seed numbers’ dependence on the soil’s chemical and physical properties. Hill slopes were not affected by water erosion, when agrophytocenoses were based on perennial grassland and also cereal–grass crop rotation, where reduced soil tillage was applied.

## 1. Introduction

Agrophytocenoses are dynamic; they change, adjusting to the annual meteorological and hydrological conditions. Human activity has a great importance as well [1,2]. An important feature for the cenopopulation to renew and exist is keeping the seeds viable as long as possible. Mature seeds germinate next season or can remain in the soil and survive unfavorable conditions there. The amount of diverse-viability seeds which are present in the soil composes the seed bank [3]. Viable seeds can lie in the ground for a very long time, and under favorable conditions germinate [4]. Plant cenopopulation age and existence depends a lot on germinating power. The seeds of plants are not equally fertile [5]. The plants of every species in a habitat compete with each other and with other plant species for survival [6]. Therefore, the plants diversely use habitat conditions and have certain biological properties to survive [7]. To successfully develop and become stronger in the habitat, plants ripen a lot of seeds and are adapted to spread them [8]. Soil erosion can deplete the weed seed bank by removing seeds and altering germination capacity [9]. High rainfall intensities on slopes produce runoff and erosion, but seeds can also be carried away by surface wash down the slope [10]. The soil runoff sediments may accumulate in low-lying areas of fields, resulting in seed burial below germination depth [9].

Seed placement within the soil matrix may play an important role in weed population dynamics. Differential incorporation of seeds of varying sizes into soil aggregates can cause differences in the microenvironment surrounding a seed and affect its fate. Weed seed distribution is variable among soil depths and aggregate sizes [11].

Under natural conditions, the seed bank is composed of seeds of varying ages, and seed density varies among soil aggregate classes. Non-aggregated seeds are generally seeds recently introduced into the soil [12].

Previous studies have demonstrated the influence of environmental factors on the species composition of weed populations [13,14]. The weed pattern is field-specific, and that spatial variation in soil properties within a field is one of several factors affecting weed patchiness [15]. Also, the management practices, soil tillage and crop type play important roles [16]. A clear separation between the effects of soil properties and the type of management is difficult for seed banks [17].

The aim of this study was to investigate the influence of different agrophytocenoses on seed numbers in the soil runoff sediments and soil seed banks’ relations to soil properties in hilly landscapes.

## 2. Materials and Methods

### 2.1. Study Areas

**Figure 1:**
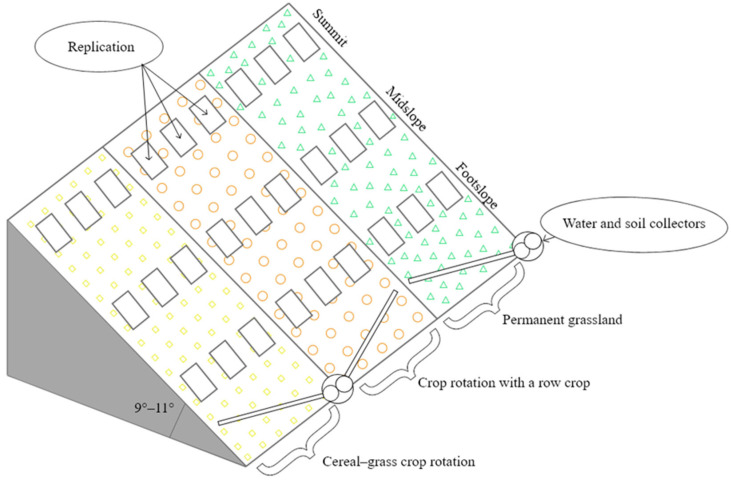
Experimental design, 2020–2022.

Evaluating the influence of environmental conditions on the soil seed bank in hilly relief, soil and plants samples have been taken from the stationary field trial established by Dr. B. Jankauskas [18,19] in Vezaiciai Branch of Institute of Agriculture of Lithuanian Research Centre for Agriculture and Forestry in 1993 (Kaltinėnai, Šilalė district, coordinates: 55°577’ N 22°482’ E). The hill exposition is southern with a steepness of 9–11°. Soil of the experiment is slightly eroded Eutric Retisol [20]. Investigations of the soil seed bank were performed in the rotations and in the permanent grassland (agrophytocenoses) in the period of 2020–2022 (Figure 1).

Factor A. Agrophytocenosis: (1) permanent grassland (PG); (2) cereal–grass crop rotation (CG); (3) crop rotation with a row crop (RC). Factor B. Parts of the hill: (1) summit; (2) mid-slope; (3) foot-slope. Indicators of the soil agrochemical and physical properties in depths of 0–5 and 5–10 cm before the experiment are represented in Table 1.

The mixture of perennial grasses for permanent grasslands, which consisted of 20% *Phleum pratense* L., 20% *Festuca rubra* L., 20% *Poa pratensis* L., 20% *Trifolium repens* L. and 20% *Lotus corniculatus* L., was sown in 1993. The grasslands were not fertilized and used.

The six-course cereal–grass crop rotation consisted of perennial grasses (2017), perennial grasses (2018), *Triticum aestivum* L. (winter crop) (2019), *Hordeum vulgare* (2020), *Triticum aestivum* (spring crop) (2021), *Hordeum vulgare* L. with undersown perennial grasses (2022).

The six-course crop rotation with a row crop consisted of *Hordeum vulgare* L. (2017), black fallow (2018), *Triticum aestivum* L. (winter crop) (2019), *Solanum tuberosum* L. (2020), *Hordeum vulgare* L. with undersown perennial grasses (2021), perennial grasses (2022).

In cereal–grass crop rotation, the primary soil tillage was reduced according to the need: shallow ploughing (10–12 cm) or shallow ploughless tillage (8–10 cm). After harvesting, the stubble was left to overwinter. In crop rotation with a row crop, the primary soil tillage was conventional ploughing (20–25 cm). Agrotechnics used in rotations are broadly described by Skuodienė et al. [21].

### 2.2. Soil Sampling and Seed Bank Analyses

Soil samples for the soil seed bank analyses were taken from each replication at 0–5 cm and 5–15 cm depths. Two kilograms of soil were collected using a drill. In total, five 100 g samples were taken out and weighed. Weighed 100 g of dry soil sample was poured into a sieve (mesh size 0.25 mm) and washed under running water, until all contents of the soil were washed out. The remaining mineral part of the soil was separated from the organic part and seeds using saturated salt solution [22]. Seed species were determined using view-magnifying optical devices, and for seed species description, A. Grigas’s [23] monograph “Lithuanian plants’ fruits and seeds” was used.

Seed number was recalculated to thousands of units per m^2^.

A = n × h × p × 100,(1)

where: A—seed number, units m^2^; n—seed number found in a sample, units; h—thickness of the examined arable layer, cm; p—soil density, g cm^3^.

Soil samples for analyses of the soil runoff sediment seeds were taken from soil and water collectors. In each agrophytocenoses, to collect the soil runoff sediments that were washed away, 2.0 m depth and 15 m diameter wells for water and soil reservoirs were installed in the foot-slope of the hill. Two reservoirs of 200 liters capacity were installed in every well. The samples were collected with a netted shovel during the vegetation period after every heavy rain that caused water flow or after reservoir filling. Dried seeds were separated from impurities; their species were determined.

Soil samples for chemical and physical analyses were taken from each replication at 0–5 and 5–15 cm depths. Soil chemical properties were determined using these methods: 

Soil acidity (pH) was measured using the potentiometric method with the extraction of 1 M of KCl (pH_KCl_), according to the International standard ISO 10390:2005 (soil quality determination of pH). In the soil, mobile P_2_O_5_ and K_2_O were determined using the Egner–Riehm–Domingo (AL) method (LVP D-07:2016), total nitrogen (N_tot_) content was determined using the Kjeldahl method, and organic carbon (C_org_) content was determined using the Dumas dry combustion method. The soil bulk density was determined with a 100 cm^3^ cylindrical drill using the Kachinsky method. Soil moisture (%) during the plant vegetation period was determined by weight method. Soil structure analysis (dry sieving)—stability of micro- and macroaggregates affected by water was determined from 0–5 and 5–15 cm depths using Savinov method. Soil texture was determined with the Fere triangle (FAO recommended method), according to the percentage of sand, silt and clay fractions in the graphical diagram.

### 2.3. Meteorological Conditions

The western region of Lithuania is strongly influenced by the maritime climate and is characterized as moderately warm and humid. In comparison with the other regions, the amount of precipitation is the highest there. During the study period, the average air temperature was 0.2°C in January and +18.1 °C in July. The average annual precipitation was 738 mm (Table 2).

### 2.4. Statistical Evaluation of the Research Data

The experimental data were assessed using multifactorial dispersion analysis method (ANOVA) [24]. Significance of the differences between the trials was evaluated with the method of dispersive analysis. The data were compared using Fisher’s protected least significant difference test (F) at a probability level of *p* < 0.05. The correlations (*r*) between experimental data were investigated using a linear regression analysis. Statistical programming software version 9.3 for all statistical analyses was SAS Enterprise [25].

Using the statistical floristic similarity coefficient (Cs), the comparison between the species composition of the soil seed bank and the soil runoff sediments was performed.

Cs = 2w/(A + B),(2)

where: w is the total number of species in both situations, A—number of species in one of two situations, B—number of species in the other situation.

## 3. Results

### 3.1. Seed Reserves in the Agrophytocenoses Soil and in Soil Runoff Sediments

The results of variance of the soil seed bank showed the significant (F_act_. = 27.8 > F_teor.01_ = 18.0) influence of different agrophytocenoses (Factor A). According to the average data of 2020–2022, the significantly smallest seed number (4036 seeds m^−2^) in the soil seed bank was determined in the soil of permanent grassland, while the highest seed number (24236 seeds m^−2^) was in the soil of cereal–grass crop rotation. In the soil seed bank of the field crop rotation with a row crop, 12915 seeds m^−2^ were found. In the soil of permanent grassland, the number of seeds was 6.0 and 3.2 times smaller compared to cereal–grass crop rotation and crop rotation with a row crop.

In persistent soil seed banks, the viable seeds composed on average 62.7–74.7%, and in temporary, they composed 62.9–66.4% of the total seed number. Irrespective of the agrophytocenosis and seed bank type, under the conditions of different soil humidity in hilly relief, the highest number of viable seeds was found in the foot-slope (66.5%), and the least number was found on the summit of the hill (61.4%). Correlation between the seed viability and the annual amount of precipitation was determined (*r* = 0.802 *p* ≤ 0.01 and 0.940 *p* ≤ 0.01, respectively: persistent and temporary seed bank).

In all investigation years, a significantly higher amount of soil (on average 3.86 t ha^−1^) was washed away in crop rotation with a row crop (Table 3). Although the difference among agrophytocenoses was significant, no correlation was determined between the amount of washed soil and seed numbers in the soil runoff sediments.

**Figure 2:**
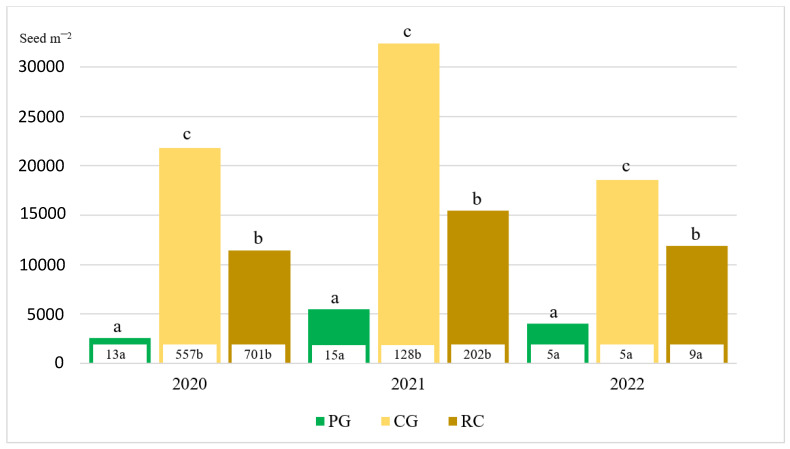
Seed number in seed bank and in soil runoff sediments (numbers in white boxes). PG—permanent grassland; CG—cereal–grass crop rotation; RC—crop rotation with a row crop. Letters a–c indicate significant (*p* ≤ 0.05) differences between the means.

Meteorological conditions (especially the amount of precipitation) were different in every year of investigation; therefore, the data of the seed number of different intensity agrophytocenoses from the water and soil collectors were evaluated separately. In 2020, 42.8 times fewer seeds were washed away from the surface of permanent grassland compared to cereal–grass crop rotation and 53.2 times fewer compared to the field crop rotation with a row crop (Figure 2).

The seeds found in the soil runoff sediments composed 0.5, 2.6 and 6.1% of the soil seed bank, respectively: in permanent grassland, cereal–grass crop rotation and field crop rotation with a row crop. Seed viability in the soil runoff sediments in agrophytocenoses was not high and diminished, respectively, in cereal–grass crop rotation (51.0%), crop rotation with a row crop (45.8%) and permanent grassland (41.9%).

The highest amount of *Chenopodium album* L. (30.7%), *Erysimum cheiranthoides* L. (23.1%) and *Myosotis arvensis* L. Hill. (15.4%) seeds were washed away from the surface of permanent grassland. The seeds of *Chenopodium album* L. (45.2%), *Fallopia convolvulus* L. (37.5%) and *Viola arvensis* Murr. (10.2%) were mostly washed away from the surfaces of the cereal–grass crop rotation and field crop rotation with a row crop (Table 4).

Comparing species composition in the soil seed bank and the soil runoff sediments, the closest in species composition (Cs = 0.69–0.71) were the field crop rotation with a row crop and the cereal–grass crop rotation, while the least similar (Cs = 0.46) was in the permanent grassland.

In 2021, it was determined that 8.5 times fewer seeds were washed away from the surface of permanent grassland compared to cereal–grass crop rotation and 13.5 times fewer compared to field crop rotation with a row crop (Figure 2). The seeds found in the soil runoff sediments composed 0.3, 0.4 and 1.3% of the soil seed bank amount, respectively, in permanent grassland, cereal–grass crop rotation and field crop rotation with a row crop. Seed viability in the soil runoff sediments in agrophytocenoses diminished, respectively, in cereal–grass crop rotation (72.7%), crop rotation with a row crop (70.0%) and permanent grassland (60.0%).

The highest amount of *Chenopodium album* L. (33.3%) and *Taraxacum officinale* F. H. Wigg. (20.0%) seeds were washed away from the surface of permanent grassland (Table 3). *Polygonum lapathifolium* L. (27.5%), *Fallopia convolvulus* L. (22.5%) and *Viola arvensis* Murr. (12.5%) seeds were mostly washed away from the surface of the cereal–grass crop rotation, while *Fallopia convolvulus* L. (27.0%), (*Chenopodium album* L. (25.4%) and *Viola arvensis* Murr. (12.6%) seeds washed from the surface of the field crop rotation with a row crop (Table 4).

Comparing species composition in the soil seed bank and the soil runoff sediments, the similarity of plant seed species in all agrophytocenoses in 2021 was lower than in 2020 (Cs = 0.56, 0.54 and 0.26, respectively, in cereal–grass crop rotation, field crop rotation with a row crop and permanent grassland).

In 2022, it was determined that, in permanent grassland and in cereal–grass crop rotation, the number of washed away seeds was diverse. In the field crop rotation with a row crop, 1.8 times more of the seeds were washed away compared to permanent grassland and cereal–grass crop rotation (Figure 2). Crops of spring barley with undersown perennial grasses were grown in the cereal–grass crop rotation, while perennial grasses of the first year of use were grown in the field crop rotation with a row crop.

The seeds found in the soil runoff sediments composed 0.1, 0.03 and 0.03% of the soil seed bank amount, respectively, in the permanent grassland, cereal–grass crop rotation and field crop rotation with a row crop. The viability of seeds found in the soil runoff sediments in agrophytocenoses decreased, respectively, in the crop rotation with a row crop (75.0%), cereal–grass crop rotation (41.7%) and permanent grassland (35.7%).

The seeds of *Viola arvensis* Murr. (80.0%) and *Polygonum lapathifolium* L. (20.0%) were washed away from the surface of the permanent grassland (Table 4). The seeds of *Fallopia convolvulus* L., *Myosotis arvensis* L. Hill., *Echinochloa crus-galli* L., *Sonchus oleraceus* L. and *Geum urbanum* L. species (20% each) were washed away from the surface of the cereal–grass crop rotation. Three species of seeds were washed away from the surface of the field crop rotation with a row crop: *Myosotis arvensis* L. Hill. (55.6%), *Sonchus oleraceus* L. (33.3%) and *Galium aparine* L. (11.1%).

Comparing species composition between the soil seed bank and soil runoff sediments, the similarity in 2022 was not high (Cs = 0.43, 0.13 and 0.18, respectively, in cereal–grass crop rotation, field crop rotation with a row crop and permanent grassland).

### 3.2. Relationships between the Seed Bank and Soil Chemical and Physical Properties

Analysing changes in the soil seed bank, correlations between the seed number and soil properties have been evaluated (soil pH, organic carbon, total nitrogen, mobile phosphorus and potassium, moisture, structure, texture) (Table 1). The chemical and physical properties of the soil changed subject to hill relief. In the downslope direction, the resources of moisture increased, organic carbon content expanded, while the soil acidity and the amount of mobile phosphorus decreased (Table 1).

Irrespective of the agrophytocenosis (Factor A), relationships between the seed number in the soil seed bank and soil chemical and physical properties were analysed in different parts of the hill (Factor B). Analysing the persistent seed bank, weak and strong correlations with soil pH, mobile phosphorus and potassium, total nitrogen and organic carbon were determined at a depth of 5–15 cm of the mid-slope of the hill and at both depths of the foot-slope of the hill, where the conditions for plant growth were better (Table 5). Also, a weak positive correlation was determined with mobile phosphorus at both depths on the summit of the hill.

Analysing the correlation data of the temporary seed bank and mobile phosphorus and potassium, as well as soil pH, similar tendencies were determined as in the assessment of the persistent seed bank. A correlation between the seed number and organic carbon, as well as total nitrogen, was not determined.

The highest amounts of mezoaggregates (1–5 mm) (20.3, 20.7 and 17.2%, respectively, in permanent grassland, cereal–grass crop rotation and field crop rotation with a row crop) were determined at a 0–15 cm depth of the foot-slope of the hill (Table 1). The same tendency was observed assessing the amount of macroaggregates (>5 mm); however they composed a smaller part than mezoaggregates (6.3, 13.7 and 7.7%, respectively, in permanent grassland, cereal–grass crop rotation and field crop rotation with a row crop). The highest amount of microaggregates (<1 mm) was determined at a 0–15 cm soil depth (from 65.6 to 96.4%). The amount of microaggregates decreased in the downslope direction.

Correlation analysis showed that seed numbers in the soil seed bank in most of cases correlated with soil aggregate–size distribution (Table 6). Analysing the persistent seed bank, weaker correlations were determined compared to the temporary seed bank, respectively, from *r* = 0.39, *p* ≤ 0.05 to *r* = 0.66, *p* ≤ 0.01 and *r* = 0.49–0.76, *p* ≤ 0.01. Positive correlations (*r* = 0.39, *p* ≤ 0.05; *r* = 0.57, *p* ≤ 0.01 and *r* = 0.68, *p* ≤ 0.01) between the soil seed number and the amount of microaggregates were determined on the summit of the hill, while negative correlations with the amount of mezoaggregates were determined on the summit and foot-slope of the hill (5–15 cm depth). Both positive and negative correlations were determined with macroaggregates, which composed a small part in the soil (0.2–17.7% of total amount of aggregates).

**Figure 3:**
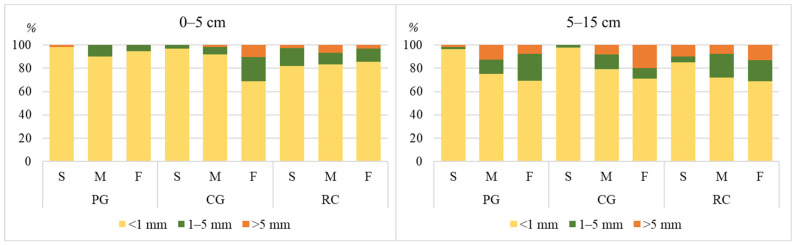
Seed (%) in soil aggregates (microaggregates (<1 mm), mezoaggregates (1–5 mm), macroaggregates (>5 mm)); Agrophytocenoses: PG—permanent grassland, CG—cereal–grass crop rotation, RC—crop rotation with a row crop; parts of the hill: S—summit, M—mid-slope, F—foot-slope.

Assessing the number of seeds washed away out of the soil aggregates at depths of 0–5 and 5–15 cm, it was determined that most of the seeds in all agrophytocenoses, respectively, 69.0–98.0% and 68.9–97.8% out of the total number of seeds washed away out of the soil, were found in microaggregates (<1 mm) (Figure 3).

In microaggregates of the permanent grassland, the number of seeds reached 87.3%, in cereal–grass crop rotation—84.3% and in field crop rotation with a row crop—79.4%.

At a 0–5 cm depth of the summit of the hill, with the lowest moisture and number of particles of clay, no correlations with the seed number in the soil seed bank were determined (Table 4). Negative weak and medium correlations with number of particles of clay were determined in other parts of the hill. Evaluating the persistent seed bank, correlations between the seed number and soil moisture were determined to be weak and medium, while, assessing the temporary seed bank, only a few significant correlations were determined.

## 4. Discussion

### 4.1. Seed Reserves in the Agrophytocenoses Soil and in the Soil Runoff Sediments

According to the research data of Grigas [23], annually, 24.2–38.6 thousands of seed per 1 m^2^ are found in arable soil, while, in non-arable soil–pasture, 3.8–27.2 thousands of seed per 1 m^2^ are found: out of which, respectively, 10.3–13.4 and 0.7–1.4 thousands are viable. Other research indicates, that, in 1 m^2^ of the arable layer (0–20 cm), there can be 20.2–71.4 thousands of seed [27]. The majority of seeds entering the seed bank come from annual weeds growing in the fields. The size of the seed bank reflects past and present field management [28]. In previous investigations, it was determined that, in cereal–grass crop rotation, where reduced soil tillage was applied, the seed number in the soil was significantly the highest. For growing row crops, the conventional soil tillage system was applied. The number of seeds in the soil was determined to be twice as small compared to the cereal–grass crop rotation, where reduced soil tillage was applied [21]. In the soil of permanent grassland, the number of seeds was determined to be significantly lower compared to cereal–grass crop rotation and crop rotation with a row crop. The examined agrophytocenoses differed in soil tillage methods. The soil of the permanent grassland was not arable. Dense turf formed in the grassland prevents the seeds from moving into the soil. The greatest part in the seed bank of permanent grassland was composed of weed seeds which are typical for arable fields, and which are viable for many years [29].

Different factors determine plant growth, development and fertility. Meteorological conditions play a key role. Relief, slope position in respect of dominating air flows and distance from the sea have the greatest significance for the precipitation distribution in Lithuania. Therefore, the average annual amount of precipitation in the west of Lithuania differs from the middle or east of Lithuania. The western regions of Lithuania are strongly affected by the marine climate (warmer winters and cooler summers compared to eastern regions); the greatest amount of precipitation here in the last 40 years reaches, on average, 923 mm per year.

According to Kinderienė, Karčauskienė [26,30], soil erosion losses and water runoff volume on the slopes of arable agricultural land generally depended on the erosion preventative capabilities of different crops, tillage technology and vegetation cover on slope, soil texture and precipitation characteristics. Climate warming processes (about 0.7 °C over more than 30 years) and a positive air temperature encouraged snow melting and runoff water flow down the slope surface, even during the cold period.

According to the long-term results, due to firm soil surface formation and coverage with the sod of grasses on the slope, permanent grassland was a constant anti-erosion protection for the slope soil (for over 30 years). The grassland was a physical and biological barrier for water runoff from the slope’s surface. Over the study time, soil erosion processes did not occur on steep slopes permanently occupied with grassland [31].

It is believed that, in 2020, due to potatoes grown in crop rotation with a row crop, the soil was cultivated up to 25–30 cm depth, and in the case of heavy precipitation (≥10 mm), the seeds present in the soil surface were carried, together with soil particles, in the downslope direction. Spring barley has been grown in cereal–grass crop rotation, where reduced soil tillage was applied (the soil cultivated up to 15 cm depth); therefore, it is likely that plant seeds have been washed away at times of heavy precipitation. In 2020, during the plant vegetation period, there were 57 days of heavy rain with ≥10 mm of precipitation per day. Permanent grassland was a constant protection of the hill slope soil from erosion [29]. Forming the grassland, the slopes of the hill were protected from water flows [32].

In 2021 it was noticed that, in crop rotation with a row crop, growing spring barley undersown with perennial grasses and using reduced soil tillage, the difference between the seeds washed away from permanent grassland was lower. Although the number of washed away seeds in cereal–grass crop rotation was quantitatively low, however, the percentage comparing agrophytocenoses remained similar. Reallocation of fallen out seeds in most cases happens due to natural processes and soil tillage [33,34,35]. Due to reduced soil tillage, the greatest part of the seeds grown in the previous year remained in the upper soil layer [36]. A lower number of the days with heavy rain also had an influence on the lower number of seeds which had been washed away. In 2021, during the plant vegetation period, there were only 16 days with heavy precipitation with ≥10 mm of precipitation per day. A similar situation was observed in 2022. A low number of days with heavy precipitation (11) had an impact on the low number of washed away seeds. Seed number in the soil runoff sediments significantly depended on the number of days with heavy precipitation during the plant vegetation period (*r* = 0.699, *p* ≤ 0.05). The viability of seeds found in the soil runoff sediments was from 45.6 to 63.6%. Lower seed viability in the soil runoff sediments compared to the seed viability of the soil seed bank could appear to be due to seed movement by the water flow.

### 4.2. Relationships between the Seed Bank and Soil Chemical and Physical Properties

Seed banks, as well as vegetation, respond to used agrotechnics and are influenced by the soil’s physical and chemical properties [37]. The soil conditions significantly affect arable seed banks directly and indirectly [17]. A direct effect occurs, for example, when waterlogged pores hinder the entrance of oxygen into the soil. This prevents germination and leads to the accumulation of seeds. An indirect effect occurs when soil conditions influence the management practice and this, in turn, affects the seed bank [17]. Mean levels were observed in soils with moderate acidic reaction and loamy-to-sandy textures. The highest seed density occurred in fields with acidic sand and/or that were seasonally wet [38].

Analysing seed bank changes, the relations between seed number and soil properties (soil pH, organic carbon, total nitrogen, mobile phosphorus and potassium, moisture, structure, texture) have been evaluated. The soil’s chemical and physical properties changed depending on hill relief. Irrespective of the agrophytocenosis (Factor A), most of the correlations between the seed numbers in soil and soil properties were determined in the foot-slope of the hill, where conditions for the growth of crop plants were better.

Other researchers indicate that organic carbon and total nitrogen amounts also have an indirect impact on the soil seed bank, influencing the productivity of maternal plants [37,39,40]. Organic carbon affects plant fertility, microbial activity, nutrient accessibility for plants, soil structure and biological activity [41]. A limited turnover of organic matter may also conserve seeds, because microbial activity can be reduced by a lack of oxygen in very wet sites or by a lack of water in sandy substrates [17].

The literature indicates that relations between the soil and seed bank strongly depend on the extent of investigation. Interaction with pH, total N concentration and C/N ratio showed up mostly on a regional scale, with diverse soil conditions. It is highlighted that phosphorus and nitrogen concentrations affected the species composition more than the number of seeds [17].

The soil’s physical characteristics determine the amount of accumulated nutrients for plants and if there is enough of water and air. In terms of agricultural, the best soil is with dominating mezoaggregates—1–5 mm diameter soil crumbs [42]. Different correlations between the seed bank and soil structural aggregates in our research can be caused by different processes, occurring in the soil due to applied soil cultivation in the agrophytocenoses, meteorological conditions (frozen ground, thaw) and diverse organic carbon sequestration in hill parts. The literature indicates that the impact of soil properties can be hardly separated from the effect of environmental factors [43]. Analysing the relations between the soil seed bank and soil structural aggregates composition, the indirect effect of soil aggregates composition can be noticed, as changes in the soil’s physical properties first of all changes the agrochemical soil quality, which directly determines the development of plants [44].

Reuss et al. [11] argue that seed distribution is variable among soil depths and aggregate sizes. The seeds present in loamy soil are usually locked into aggregates. Seeds were commonly found associated with aggregates larger than 9 mm. Seed viability in aggregates was determined to be lower [11]. However, in our investigation, the great seed number in microaggregates could be an error of calculation of the carried out analysis. Soil aggregates, especially the large ones (macro- and mezoaggregates) could fall to pieces due to mechanical influence while carrying out the analysis, while the seeds, together with loosened single soil particles, could get into fractions of the smallest aggregates. Veršulienė [44] indicates that one of the most important factors influencing aggregate stability is soil organic carbon. 

## 5. Conclusions

Soil tillage intensity in the agrophytocenoses has a significant impact on the seed bank in hilly relief. Significantly, the lowest seed number was determined in the soil of permanent grassland (non-arable soil for more than 30 years), while the highest seed number was determined in the soil of cereal–grass crop rotation, where reduced soil tillage was applied.

Seed number in the soil runoff sediments significantly depended on the number of days with heavy precipitation during the plant vegetation period, as well as on the plant communities appearing in certain agrophytocenoses and the biological traits of plants.

The seeds found in the soil runoff sediments composed on average 0.3, 0.1 and 2.4% of the soil seed bank amount, respectively, of permanent grassland, cereal–grass crop rotation and crop rotation with a row crop. The viability of seeds found in the soil runoff sediments in agrophytocenoses decreased, respectively, in the crop rotation with a row crop (63.6%), cereal–grass crop rotation (55.1%) and permanent grassland (45.6%).

The highest amount of *Viola arvensis* Murr. (29.2%) and *Chenopodium album* L. (21.3%) seeds were washed away from the permanent grassland; the highest amount of *Fallopia convolvulus* L. (26.7%) and *Chenopodium album* L. (16.7%) seeds were washed away from cereal–grass crop rotation; and *Chenopodium album* L. (24.8%), *Myosotis arvensis* L. (20,0%) and *Fallopia convolvulus* L. (19.1%) seeds were washed away from the crop rotation with a row crop.

Irrespective of the agrophytocenosis, most of correlations between the seed number and soil properties have been determined in the foot=slope of the hill, where the conditions for the growth of crop plants were better (higher resources of soil moisture, organic carbon, nutrients and lower soil acidity).

In areas with a high erosion risk, it is very important to form an agrophytocenosis that will be productive on hilly terrain and can protect the soil from erosion effectively [45]. Hill slopes were not affected by water erosion when agrophytocenoses were based on perennial grassland and also cereal–grass crop rotation, where reduced soil tillage was applied.

## Figures and Tables

**Table 1:** Agrochemical and physical properties of the arable (0–15 cm) soil depth.

Soil Properties	Parts of the Hill					
	Summit		Mid-Slope		Foot-Slope	
	0–5 cm	5–15 cm	0–5 cm	5–15 cm	0–5 cm	5–15 cm
Permanent grassland						
Soil acidity ^1^ (pH_KCl_)	5.5	5.8	6.4	6.0	5.1	4.9
Mobile P_2_O_5_^1^ (mg/kg)	52.7	39.7	96.0	46.3	38.3	14.0
Mobile K_2_O ^1^ (mg/kg)	253.7	138.3	250.7	132.3	329.7	154.0
Total N ^1^ (%)	0.132	0.096	0.150	0.124	0.162	0.101
Organic C ^1^ (%)	1.12	0.97	1.54	1.17	1.65	1.12
Soil density ^1^ Mg m^−3^	1.00	1.12	0.76	0.93	0.81	1.07
Soil moisture ^2^ (%)	21.4–25.5	14.5–18.0	22.3–42.7	22.3–23.4	23.0–30.7	19.5–20.7
Microaggregates (<1 mm) (%)	90.2	90.2	91.4	89.2	77.9	69.0
Mezoaggregates (1–5 mm) (%)	9.3	9.0	7.5	9.3	19.0	21.5
Macroaggregates (>5 mm) (%)	0.5	0.8	1.1	1.5	3.1	9.5
Cereal–grass crop rotation						
Soil acidity ^1^ (pH_KCl_)	5.6	5.4	5.3	5.1	5.1	5.1
Mobile P_2_O_5_^1^ (mg/kg)	192	201	165	168	149	148
Mobile K_2_O ^1^ (mg/kg)	209	112	198	98	223	107
Total N ^1^ (%)	0.078	0.077	0.097	0.096	0.106	0.101
Organic C ^1^ (%)	0.9	0.8	1.1	1.0	1.1	1.0
Soil density ^1^ Mg m^−3^	1.34	1.36	1.35	1.35	1.27	1.33
Soil moisture^2^ (%)	12.1–16.1	11.9–14.7	15.4–21.2	14.1–19.4	17.8–21.8	16.9–20.6
Microaggregates (<1 mm) (%)	97.2	95.7	88.6	84.4	69.1	62.1
Mezoaggregates (1–5 mm) (%)	2.6	3.9	8.6	9.4	21.2	20.2
Macroaggregates (>5 mm) (%)	0.2	0.4	2.8	6.2	9.7	17.7
Crop rotation with a row crop						
Soil acidity ^1^ (pH_KCl_)	6.4	6.6	5.4	5.7	5.2	5.4
Mobile P_2_O_5_^1^ (mg/kg)	211.7	213.7	174.3	174.0	163.7	140.0
Mobile K_2_O ^1^ (mg/kg)	181.7	112.7	225.0	103.7	207.3	116.3
Total N ^1^ (%)	0.072	0.057	0.084	0.082	0.091	0.085
Organic C ^1^ (%)	0.65	0.68	0.82	0.82	0.94	0.82
Soil density ^1^ Mg m^−3^	1.32	1.38	1.30	1.30	1.38	1.44
Soil moisture ^2^ (%)	13.0–15.7	13.3–14.6	15.3–16.6	15.4–16.4	18.2–20.2	17.5–18.9
Microaggregates (<1 mm) (%)	98.6	94.1	86.2	79.6	81.6	68.7
Mezoaggregates (1–5 mm) (%)	1.2	3.9	9.9	12.6	15.2	19.1
Macroaggregates (>5 mm) (%)	0.2	2.0	3.9	7.8	3.2	12.2

^1^ Reprinted from Skuodienė et al. [21]; ^2^ Min–max. values during the growing season.

**Table 2:** Weather conditions during the study period, 2020–2022.

	2020	2021	2022	SCN
Annual mean temperature °C	8.6	7.0	7.4	6.7
Growing season’s mean air temperature °C	12.9	12.7	12.6	12.2
Total annual precipitation mm	756	738	719	789
Growing season’s total precipitation mm	394	522	420	481

**Table 3:** Indicators of soil erosion processes of slope soil using different land-use systems.

Agrophytocenosis	Runoff Coefficient ^1^	Soil Loss Reduction Effectiveness (SLRE) ^1^	Soil Erosion Losses t ha^−1^
	1995–2012	1995–2012	2020–2022
Permanent grassland	0.36	99.30	0.00a
Cereal–grass crop rotation	0.54	89.30	0.01a
Crop rotation with a row crop	0.67	0.00	3.86b

^1^ Reprinted from Kinderienė, Karčauskienė [26]; Letters a and b indicate significant (*p* ≤ 0.05) differences between the means.

**Table 4:** Species composition of seeds in soil runoff sediments (%).

Botanic Family	Plant Species	Agrophytocenosis								
		PG			CG			RC		
		2020	2021	2022	2020	2021	2022	2020	2021	2022
Asteraceae	*Cirsium arvense* (L.) Scop.				0.7	2.5		0.7		
	*Sonchus asper* L.		6.8							
	*Sonchus oleraceus* L.	7.7	13.3		0.7		20.0	1.4		33.3
	*Tripleurospermum perforatum* M. Lainz				0.2					
Boraginaceae	*Myosotis arvensis* L.	15.4			0.7	5.0	20.0	1.1	3.2	55.6
Brassicaceae	*Erysimum cheiranthoides* L.	23.1			0.2					
Caryophyllaceae	*Stellaria media* (L.) Vill.								4.8	
	*Spergula arvensis* L.		13.3		1.3	7.5		1.6	1.6	
	*Taraxacum officinale* F. H. Wigg.		20.0			2.5				
Chenopodiaceae	*Chenopodium album* L.	30.7	33.3		45.2	5.0		49.1	25.4	
Fabaceae	*Trifolium repens* L.					5.0				
Lamiaceae	*Lamium purpureum* L.				1.1	5.0		0.7	1.6	
Poaceae	*Echinochloa crus-gali* L.					5.0	20.0		3.2	
	*Setaria viridis* P. B.				0.4			0.3	9.5	
Polygonaceae	*Fallopia convolvulus* (L.) A. Löve.	7.7	13.3		37.5	22.5	20.0	30.4	27.0	
	*Polygonum lapathifolium* L.	7.7		20.0	1.8	27.5		1.7	7.9	
	*Rumex acetosella* L.							0.6		
	*Rumex crispus* L.								3.2	
Rosaceae	*Geum urbanum* L.						20.0			
Rubiaceae	*Galium aparine* L.									11.1
Violaceae	*Viola arvensis* Murr.	7.7		80.0	10.2	12.5		12.4	12.6	
The number of species		7	6	2	12	11	5	11	11	3
Annual monocotyledonous,%		0	0	0	0.4	5.0	20.0	0.3	12.7	0
Annual dicotyledonous,%		92.3	59.9	100.0	98.2	85.0	40.0	97.0	84.1	66.7
Perennial monocotyledonous,%		0	0	0	0	0	0	0	0	0
Perennial dicotyledonous,%		7.7	40.1	0	1.4	10.0	40.0	2.7	3.2	33.3

PG—permanent grassland; CG—cereal–grass crop rotation; RC—crop rotation with a row crop.

**Table 5:** Correlations between the seed number in the soil seed bank and soil chemical properties (*n* = 27).

Properties	Depth cm	Persistent Seed Bank			Temporary Seed Bank		
		Parts of the Hill			Parts of the Hill		
		Summit	Mid-Slope	Foot-Slope	Summit	Mid-Slope	Foot-Slope
Soil acidity (pH_KCl_)	0–5					−0.52 **	0.66 **
	5–15		−0.54 **	−0.55 **		−0.75 **	
Mobile P_2_O_5_ (mg/kg)	0–5	0.38 *		0.58 **			0.48 *
	5–15	0.42 *	0.49 **	0.58 **	0.49 **	0.84 **	0.63 **
Mobile K_2_O (mg/kg)	0–5					−0.42 *	−0.48 *
	5–15			−0.50 **	−0.47 *	−0.52 **	−0.43 *
Total N (%)	0–5			−0.45 *			
	5–15			−0.41 *			
Organic C (%)	0–5			−0.52 **			
	5–15						

*, ** the least significant difference at *p* ≤ 0.05 and *p* ≤ 0.01, respectively.

**Table 6:** Correlations between the seed number in the soil seed bank and soil physical properties (*n* = 27).

Properties	Depth cm	Persistent Seed Bank			Temporary Seed Bank		
		Parts of the Hill			Parts of the Hill		
		Summit	Mid-Slope	Foot-Slope	Summit	Mid-Slope	Foot-Slope
Microaggregates (<1 mm)	0–5	0.39 *		−0.46 *			
	5–15	0.57 **			0.68 **	−0.62 **	
Mezoaggregates (1–5 mm)	0–5						
	5–15	−0.49 *		−0.66 **	−0.55 **		−0.74 **
Macroaggregates (>5 mm)	0–5	−0.56 **		0.60 **	−0.58 **		0.49 **
	5–15		0.43 *			0.76 **	
Moisture%	0–5		−0.40 *	−0.48 *			
	5–15	−0.47 *	−0.54 **	−0.65 **	−0.45 *		−0.39 *
Clay%	0–5		−0.58 **	−0.45 *		−0.64 **	−0.39 *
	5–15	−0.58 **		−0.65 **	−0.71 **	−0.40 **	−0.68 **

*, ** the least significant difference at *p* ≤ 0.05 and *p* ≤ 0.01, respectively.

## Data Availability

Data is contained within the article.
